# Assay validation for the assessment of adipogenesis of multipotential stromal cells—a direct comparison of four different methods

**DOI:** 10.1016/j.jcyt.2012.07.001

**Published:** 2013-01

**Authors:** Andrew Aldridge, Dimitrios Kouroupis, Sarah Churchman, Anne English, Eileen Ingham, Elena Jones

**Affiliations:** 1 Institute of Molecular and Cellular Biology, Institute of Medical and Biological Engineering, Faculty of Biological Sciences, University of Leeds, Leeds, West Yorkshire, United Kingdom; 2 Academic Unit of Musculoskeletal Disease, Leeds Institute of Molecular Medicine, University of Leeds, Leeds, West Yorkshire, United Kingdom

**Keywords:** adipogenesis, flow cytometry, multipotential stromal cells, Nile red

## Abstract

**Background aims:**

Mesenchymal stromal cells (MSCs) are regenerative and immuno-privileged cells that are used for both tissue regeneration and treatment of severe inflammation-related disease. For quality control of manufactured MSC batches in regard to mature fat cell contamination, a quantitative method for measuring adipogenesis is needed.

**Methods:**

Four previously proposed methods were validated with the use of bone marrow (BM) MSCs during a 21-day *in vitro* assay. Oil red staining was scored semiquantitatively; peroxisome proliferator activated receptor-γ and fatty acid binding protein (FABP)4 transcripts were measured by quantitative real-time polymerase chain reaction; FABP4 protein accumulation was evaluated by flow cytometry; and Nile red/4′,6-diamidino-2-phenylindole (DAPI) ratios were measured in fluorescent microplate assay. Skin fibroblasts and MSCs from fat pad, cartilage and umbilical cord were used as controls.

**Results:**

Oil red staining indicated considerable heterogeneity between BM donors and individual cells within the same culture. FABP4 transcript levels increased 100- to 5000-fold by day 21, with large donor variability observed. Flow cytometry revealed increasing intra-culture heterogeneity over time; more granular cells accumulated more FABP4 protein and Nile red fluorescence compared with less granular cells. Nile red increase in day-21 MSCs was ∼5- and 4-fold, measured by flow cytometry or microplate assay, respectively. MSC proliferation/apoptosis was accounted through the use of Nile red/DAPI ratios; adipogenesis levels in day-21 BM MSCs increased ∼13-fold, with significant correlations with oil red scoring observed for MSC from other sources.

**Conclusions:**

Flow cytometry permits the study of MSC differentiation at the single-cell level and sorting more and less mature cells from mixed cell populations. The microplate assay with the use of the Nile red/DAPI ratio provides rapid quantitative measurements and could be used as a low-cost, high-throughput method to quality-control MSC batches from different tissue sources.

## Introduction

The capacity of multipotential stromal cells (MSCs) to differentiate into a range of cell types including chondrocytes, tenocytes, adipocytes and osteoblasts is well established (1–3). Typically, MSCs are isolated from bone marrow (BM) aspirates taken from the iliac crest. Although MSCs represent only a small fraction of nucleated cells in the BM aspirates (between 0.001–0.01%), they can expanded with high efficiency (3–5), representing a rapidly expandable, easy to access source of multipotent cells to treat a wide variety of conditions through tissue engineering and cell therapy. Another feature that makes MSCs desirable for use in regenerative medicine is the reported immunoprivileged nature of these cells and their ability to reduce inflammation and exhibit immunosuppressive effects (6–8). This has led to the use of allogeneic MSCs in regenerative medicine applications (9,10). Much of the research in the immunological properties of MSCs, however, has been carried out on undifferentiated MSCs, and although there are some reports on immune-marker expression on MSCs after short-term differentiation toward bone, fat or cartilage tissue (11), little is known with respect to the immunological properties of MSCs after longer-term differentiation, including longer-term adipogenic differentiation for the therapeutic applications aimed at plastic and reconstructive surgery.

The differentiation of MSCs into adipocytes presents an interesting system for the study of their post-differentiation immunological properties. Mature adipose cells have been shown to secrete hormones termed “adipokines,” such as leptin, resistin and adiponectin (12). These adipokines are known to exert potent immunomodulatory effects on cells of the immune system (12,13). At the same time, adipocytes produce pro-inflammatory cytokines (tumor necrosis factor-α and interleukin-6) and chemokines such as monocyte chemoattractant protein-1 (MCP)-1 (12,14). Contamination of therapeutic MSC batches with preadipocytes and adipocytes, particularly those derived from lipoaspirates, may therefore pose additional safety considerations in the treatment of Crohn disease (15,16) and in reconstructive surgery (17).

To study the immunological properties of MSCs during the process of adipogenic differentiation *in vitro* and after implantation *in vivo*, a robust method to quantify the achieved level of adipogenesis is required. Various methods have been used previously to evaluate MSC adipogenesis, such as quantitative real-time polymerase chain reaction (q-PCR) for adipogenesis-related transcripts (18–22), flow cytometry (18,20,23,24), oil red O staining (3,4,19,25,26) and Nile red staining (18,20,23,27). However, a comparative evaluation of these different methods with the use of MSCs from the same donors has not yet been performed. The aim of this study was to directly compare these methods to (i) establish the most robust method for measuring the extent of adipogenesis achieved and (ii) evaluate cell-to-cell heterogeneity within the same MSC batch, thus allowing evaluation of direct effects of MSC adipogenic commitment on their immunoregulatory properties.

## Methods

### Isolation and expansion of MSCs and control skin fibroblasts

Unless otherwise stated, MSCs used in this study were isolated from BM aspirates. Human BM was obtained from the iliac crest of four healthy donors (median age, 24; range, 8–69 years), and MSC cultures were established according to Pittenger *et al.* (3). Briefly, mononuclear cells (MNCs) were isolated from BM through the use of Lymphoprep (Nycomed Pharma, Oslo, Norway), and plastic-adherent cells were grown in NH Expansion medium (Miltenyi Biotech, Surrey, UK) in 25-cm^2^ tissue culture flasks (Corning, Flintshire, UK) at 37°C 5% (v/v) CO_2_ in air to passage 2 (p2). In addition, MSCs were grown from knee cartilage and fat pads of patients with osteoarthritis (n = 3; median age, 72.5 years; range, 69–77 years), as described previously (25), or from umbilical cord tissue (n = 3), according to methods described by Majore *et al.* (28) and Karahuseyinoglu *et al.* (29).

Human skin fibroblasts were obtained from the American Type Culture Collection (ATCC) (Teddington, UK) and were expanded in complete Dulbecco modified Eagle's medium (DMEM, Invitrogen, Paisley, UK) with 10% fetal calf serum (FCS), 50 U.mL^−1^ penicillin/streptomycin and 2 mmol/L L-glutamine (all from Sigma, Poole, UK). Ethical permission to use human tissue samples was obtained from Leeds Teaching Hospitals NHS Trust.

### Adipogenic differentiation of MSCs and fibroblasts

Adipogenic medium was made by supplementing complete DMEM with 10% v/v FCS, 10% v/v horse serum (Stem Cell Technologies, Sheffield, UK), 0.5 mmol/L isobutylmethylxanthine (IBMX), 0.06 mmol/L indomethacin, 50 pmol/L hydrocortisone, 2 mmol/L L-glutamine (all from Sigma) and antibiotics, as previously described (3,4,11,30). Adipogenic differentiation assays were performed in 24- and 48-well plates, with the use of constant cell seeding density of 4 × 10^4^ cells per cm^2^. Adipogenic medium was changed twice weekly (one-half media changes) during a 21-day time course, and samples were taken for analysis at different time points as specified below.

### Oil red staining for semiquantitative scoring of adipogenesis

Before staining, cells were washed twice with PBS and fixed in 10% (v/v) formalin (Biostain Ready Reagents, Manchester, UK). After fixation and two further washes in PBS, oil red solution [0.5% oil red (w/v) (Sigma) in isopropanol] was added for 10 min. After incubation at room temperature (RT), oil red was removed, and cells were washed twice in PBS and stained with Harris hematoxylin (Surgipath, Bretton, UK) for 45 seconds. Subsequently, cells were washed in tap water and allowed to dry before viewing.

**Figure 1:**
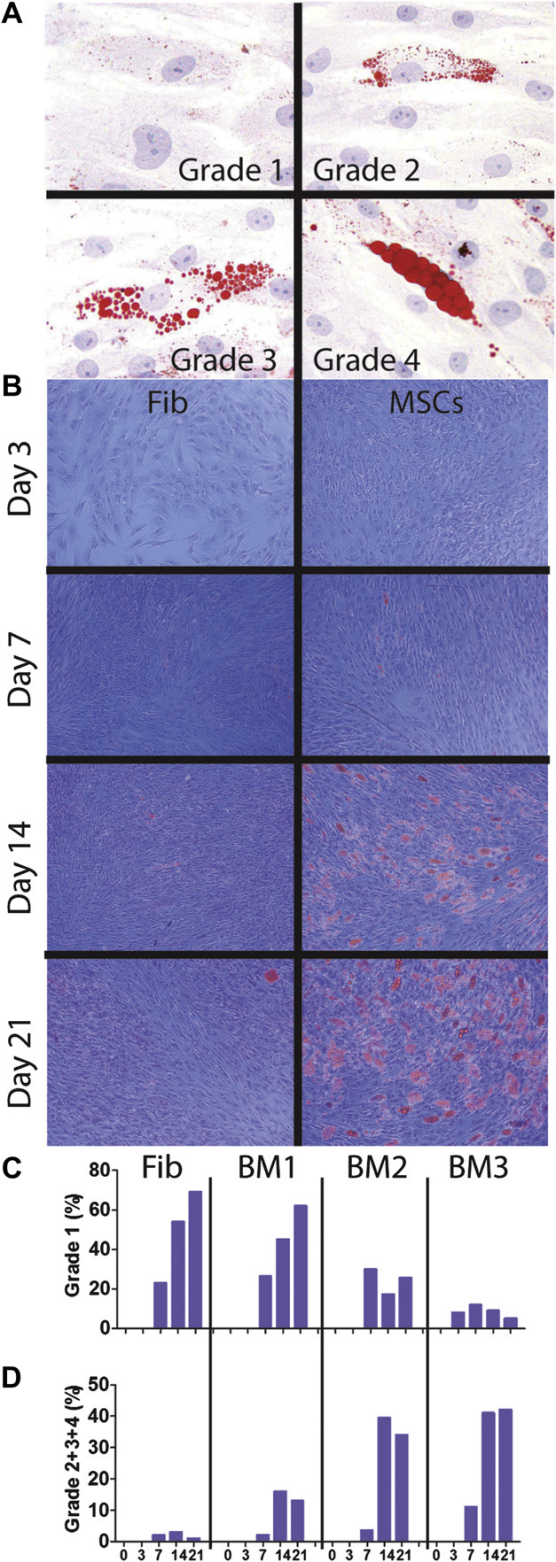
Semiquantitative scoring of adipogenesis with the use of oil red. (A) Visual grading scheme on the basis of lipid droplet accumulation: microphotographs of representative cells assigned to grades 1–4. Grades represent the proportion of cell cytoplasm occupied by lipid droplets. (B) Adipogenesis time course: fibroblasts (left panels) and BM MSCs (right panels) were differentiated over 21 days into fat lineage, and microphotographs were taken from a central area of a representative triplicate well. (C) Percentage of grade 1 cells. (D) Percentage of cells of grades 2 and above. BM1-3 represents the three different donors, and the percentage shown is the mean value from triplicate wells for each donor. *X*-axis numbers represent days of culture. Original magnifications: ×400 for A and ×100 for B.

The amount of fat in individual cells was scored semiquantitatively through the use of a grading scheme developed by English *et al.* (25). The level of adipogenesis was described by ranking 500 cells in a middle area of the well on their fat content. Ranks were allocated on the basis of the proportion of cytoplasm occupied by fat globules: grade 1: 0–24%; grade 2: 25–49%; grade 3: 50–74%; grade 4: 75–100% (Figure 1A). Subsequently, a relative percentage of cells assigned to each grade was calculated for triplicate wells and averaged.

### Quantitative real-time PCR for peroxisome proliferator activated receptor-γ and fatty acid binding protein 4 gene expression

Peroxisome proliferator activated receptor (PPAR)-γ and particularly fatty acid binding protein (FABP)4 (31) have been previously used as common molecular markers of MSC adipogenesis (18,20,22). RNA was extracted and complementary (c)DNA was synthesized with the use of Superscript II reverse transcriptase (Invitrogen). Q-PCR was performed with the use of an AB Prism 7900HT sequence detection system (Applied Biosystems, Warrington, UK) in the presence of SYBR-green. Primers were designed with the use of Primer Express 2 (Applied Biosystems) and supplied by MWG Biotech (Ebersberg, Germany). Expression was normalized through the use of the reference gene GAPDH. Primer concentrations used were PPAR-γ F; cac aag aac aga tcc agt ggt tg (500 nmol/L), PPAR-γ R; gag gct tat tga gag ctg agt ctt ct (500 nmol/L), FABP4 F; cca taa aga gaa aac gag agg atg at (1000 nmol/L), FABP4 R; tgg aag tga cgc ctt tca tga (1000 nmol/L), GAPDH F; aac agc gac acc cac tcc tc (500 nmol/L), GAPDH R; cat acc agg aaa tga gct tga caa (500 nmol/L); all primer pairs were tested and found to be equally efficient. MSCs from three different donors were used, and for each time point the mean Ct of three technical replicates of each gene of interest was used to calculate the relative gene expression.

### Flow cytometry for FABP4 and Nile red staining

For Nile red staining, cells were harvested by treatment with 0.5% trypsin/EDTA (Invitrogen), washed in PBS and fixed with 10% (v/v) formalin in PBS. Stock Nile red (Sigma) solution (0.1 mg.mL^−1^, prepared in methanol) was added to cells at a final concentration of 0.05 μg mL^−1^ (1:2000 dilution in PBS) (17) and incubated at 4°C for 20 min. Cells were washed in BD FACSflow before flow cytometry was carried out with the use of a FACScan. Nile red fluorescence emission in adipogenically differentiated MSCs was detected on FL2 channel (bandpass filter, 585 ± 42 nm).

FABP4 protein was detected with the use of a purified polyclonal goat anti-human FABP4 antibody (IgG, R & D Systems, Abingdon, UK) at 1:20 dilution in PBS. For intracellular staining with FABP4, trypsinized cells were initially fixed in 100 μL of Intrastain reagent A (Dako, Cambridge, UK). FABP4 antibody was added together with 100 μL of permeabilizing reagent/Intrastain reagent B (Dako) for 15 min at RT in the dark. After two washes with PBS, cells were incubated with biotinilated rabbit anti-goat secondary antibody (1:400 dilution in PBS, Dako) for 15 min at RT in the dark. After two more washes in PBS, the binding was visualized with the use of streptavidin-FITC (1:50 dilution, BD Sciences). After two final washes, the fluorescence was analyzed on FL1 channel (bandpass filter, 530 ± 30 nm), with 10000 events being collected in each sample.

### Flow cytometry for MSC marker expression

The following antibodies were used to test MSC marker expression on undifferentiated and day-16 adipogenically differentiated BM MSCs (n = 2) and skin fibroblasts (n = 1): CD73-PE, CD90-PE, CD166-PE, CD44-PE (all from BD, Oxford, UK), CD105-PE, CD13-FITC, CD31-FITC (all from Serotec, Kidlington, UK) and CD45-FITC (Dako, Ely, UK). All isotype controls were from Serotec, and extended phenotyping of polyclonal and clonal cultures was performed as previously described (32).

### Measure of adipogenic differentiation using Nile red/4′,6-diamidino-2-phenylindole (DAPI) staining

MSCs and fibroblasts were seeded in a 48-well plate (Nunc, Loughborough, UK) and cultured for 21 days in adipogenic medium. On days 0, 3, 7, 14 and 21, the level of adipogenesis was determined with the use of a luminescent plate reader (LB940 Multilabel Reader Mithras; Berthold Technologies), with each condition having six replicates. At each time point, the medium was removed; wells were washed twice with PBS and fixed with 200 μL of formalin (10% v/v in PBS) for 30 min at RT. Subsequently, wells were washed twice with PBS and the background fluorescence was read in 200 μL of PBS with the use of DAPI (355/460) and Nile red (485/540) filter sets as per the manufacturer's instructions (27,33). These filter sets were used because they provided the closest match to DAPI (excitation at 358 nm and emission at 461 nm) and Nile red (excitation at 450–500 nm and emission at >528 nm) (23,33).

Wells were spot-read in a 10 × 10 pattern per well. After background reading, the PBS was removed and 200 μL of saponin (Sigma, Poole, Dorset, UK) (0.2% w/v in PBS) was added along with DAPI (Invitrogen) at a final concentration of 1 μg.mL^−1^ and Nile red at a final concentration of 1 μg.mL^−1^ (33). The plate was then wrapped in foil and incubated at RT for 15 minutes. After three washes with PBS, 200 μL of PBS was added and plates were again read as above.

To calculate the Nile red:DAPI ratio, the background fluorescence was first subtracted from the readings. The mean fluorescent reading for six replicates was calculated for both Nile red and DAPI; Nile red:DAPI ratio was then established and normalized to day 0 (undifferentiated cells). Fluorescent microscopy images were taken with an Olympus IX71 inverted microscope using CellˆB image capture software (Olympus).

### Statistical analysis

To determine whether the methods used for measuring adipogenic progression were comparable, *R* values and two-tailed *P* values were calculated by means of Spearman correlation in Graphpad Prism 5. Standard deviations were also calculated by means of Graphpad Prism 5.

## Results

### Semiquantitative scoring of adipogenesis of MSCs with the use of oil red staining

The most common staining for adipogenically differentiated MSCs (oil red staining) was initially used and quantified by means of a visual grading system (25) whereby the level of adipogenic progression in 500 cells in a central area of the well was ranked from 1–4 on the basis of the proportion of cytoplasm occupied by fat in each cell (Figure 1A). Subsequently, a relative percentage of cells assigned to each grade was calculated for triplicate wells and averaged. In these experiments, MSCs from three BM donors and negative control skin fibroblasts were grown in adipogenic medium for 21 days (Figure 1B), and scoring was performed on days 0, 3, 7, 14 and 21 after induction (Figure 1B–D).

As seen in Figure 1C, fibroblasts gradually accrued grade 1 levels of fat content; however, they were unable to progress to higher grades in fat accumulation (Figure 1D). In all MSCs, differentiation had begun as a gradual accumulation of grade 1 cells (Figure 1C). In contrast to fibroblasts, however, MSCs continued to amass fat in their cytoplasm and by days 14–21 contained cells with high fat content (grades 2–4, Figure 1D).

These experiments showed that although fibroblasts were clearly inferior to MSCs, they had some capacity for adipogenesis. Furthermore, adipogenic progression in MSCs from the same donor was heterogeneous, with some cells in the cultures progressing to grades 3–4 and others remaining at grade 1. Finally, donor-to-donor differences in the rates and the amounts of adipogenesis in MSCs were also observed, with BM1 being more “resistant” to adipogenesis, compared with the other two donors (the latter easily progressed to grades 3–4). Altogether, these data showed that more quantitative methods of measuring adipogenesis are needed to account for these differences.

### Quantitative changes in PPAR-γ and FABP4 messenger (m)RNA expression in MSCs undergoing adipogenesis

**Figure 2:**
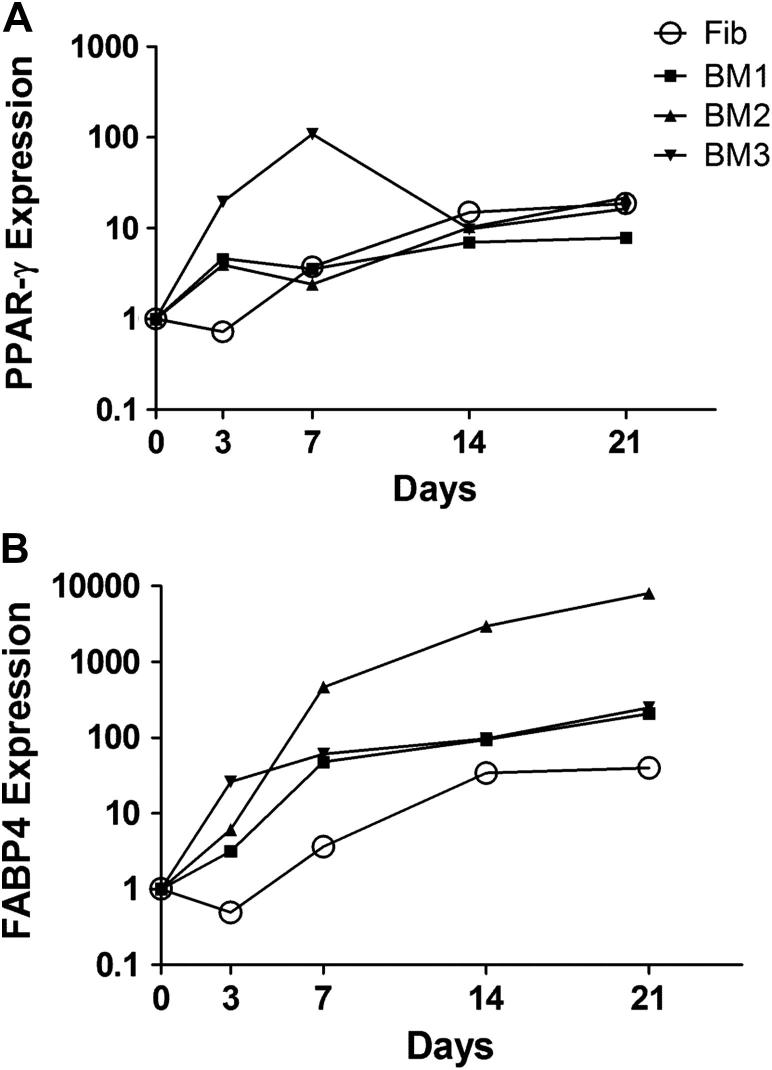
Monitoring adipogenic progression of MSCs and fibroblasts with the use of q-PCR. MSCs and fibroblasts were cultured in adipogenic medium for 21 days. Expression of (A) PPAR-γ and (B) FABP4 was determined on days 0, 3, 7, 14 and 21. Relative levels of gene expression were normalized to reference gene GAPDH and displayed as fold increase over day 0. Each data point represents the mean of three replicates.

Adipogenesis-specific PPAR-γ and the late marker of adipogenesis, FABP4, have been previously shown to closely reflect adipogenic progression of MSCs (18,20,22,34). PPAR-γ and FABP4 mRNA levels were next determined in adipogenically differentiated MSCs and correlated to morphological fat accumulation within the cells. When normalized to GAPDH, donor-to-donor differences in PPAR-γ expression levels in MSCs on day 0 were considerable (7-fold); therefore relative gene expression data for days 3, 7, 14 and 21 after induction were further normalized to their baseline levels in undifferentiated cells (day 0) (Figure 2).

In fibroblasts, PPAR-γ gene expression increased gradually to approximately 30-fold on day 21. PPAR-γ gene expression in MSCs increased gradually to approximately the same levels, with greater donor variability observed at earlier time points (Figure 2A). FABP4 gene expression in fibroblasts mirrored that of PPAR-γ (Figure 2B). FABP4 gene expression in MSCs was above that of fibroblasts at all time points and increased by 2–3 orders of magnitude by day 21.

### Monitoring FABP4 protein accumulation and its correlation with Nile red staining by flow cytometry

**Figure 3:**
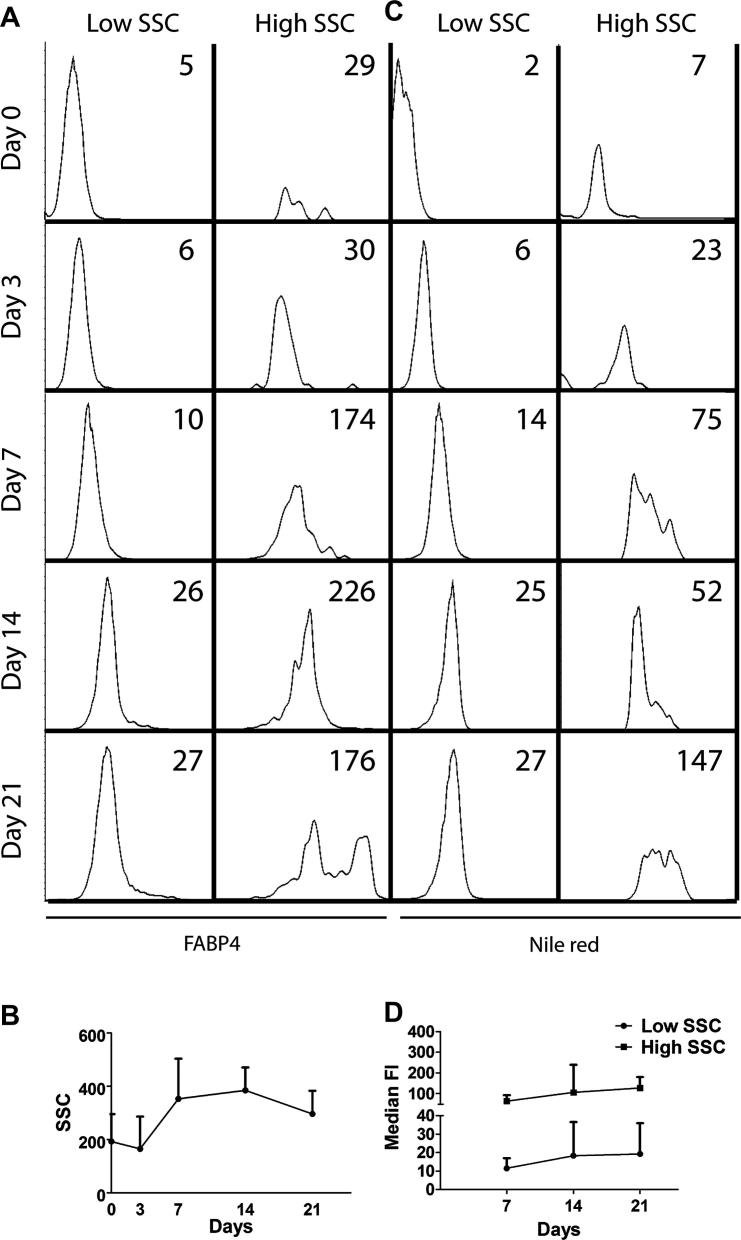
Monitoring adipogenic progression of MSCs with the use of flow cytometry for FABP4 and Nile red. (A) Representative histogram plots for FABP4 staining. (B) The increase in SSC characteristics for the whole population of cells as the time course progresses; error bars represent standard deviation of the mean for three donors tested. (C) Representative histogram plots for Nile red staining. (D) Nile red fluorescence of both populations of gated low- and high-SSC cells, demonstrating a parallel increase throughout the time course. Days 0 and 3 are omitted because of very low frequency of high-SSC early in differentiation. Numbers in the top right corners of histograms represent median fluorescence intensities. FI is fluorescent intensity.

FABP4 has been documented as one of the best markers of adipogenesis measured by q-PCR (31); however, FABP4 protein accumulation in adipogenically driven MSCs by flow cytometry has not been reported and was next investigated (Figure 3A).

First, flow cytometry has revealed some interesting changes in MSC scatter characteristics as a result of their differentiation towards the adipogenic lineage. At the later stages of differentiation, an accumulation of more granular cells (high side scatter [SSC], possibly reflecting fat globuli) was observed. As expected, FABP4 protein gradually accumulated in MSCs, and its levels were always higher in more granular cells (high-SSC) compared with less granular cells (low-SSC) (Figure 3A). Differences in granularity/SSC were, however, too small (<2-fold by day 14) to be considered as a robust measure of adipogenesis (Figure 3B). Furthermore, a drop in the proportion of high-SSC cells by day 21 was observed (Figure 3B) and could be explained by their loss during centrifugation steps because they were expected to be more buoyant.

The mean level of FABP4 protein accumulation in MSCs on day 21 was 6-fold stronger compared with day 0. These data clearly confirmed the existence of cell-to-cell heterogeneity within the same adipogenically driven MSC culture, first observed by morphological analysis with oil red. Flow cytometry for FABP4+ cells and semiquantitative scoring with oil red displayed excellent correlation (*r* = 0.989, *P* ≤ 0.0001).

FABP4 staining for flow cytometry requires cell permeabilization and multiple centrifugation steps; therefore a simple cytometric method of staining with Nile red, a dye that binds to intracellular lipids, was next attempted (23,35–37). Similar trends were observed when Nile red staining was used instead of FABP4 (Figure 3C). Nile red accumulation in low-SSC cells and high-SSC cells increased in parallel (Figure 3D). The average overall Nile red content in MSCs on day 21 was 5-fold stronger compared with day 0. With the use of the same donor samples, Nile red dye and intracellular FABP4 antibody labeling demonstrated excellent correlation (*r* = 0.958, *P* < 0.0001). Nile red–based flow cytometry also showed good correlation with morphological scoring by oil red (*r* = 0.952, *P* < 0.0001) for all donors and all time points tested.

No significant accumulation of FABP4+ or Nile red+ cells was observed during adipogenic differentiation of fibroblasts (data not shown). The expression of several MSC surface markers (CD73, CD105, CD90, CD44, CD166, CD13) and non-MSC controls [CD45, CD31 (38)] was next investigated on day-16 adipogenic MSC cultures and fibroblasts and compared with their baseline levels in undifferentiated cells. No significant differences in the expression of any markers were found between MSCs and fibroblasts before differentiation (CD73^+^CD105^+^CD90^+^CD44^+^CD166^+^CD13^+^CD45^−^CD31^−^), consistent with previous reports (39), as well as between day-0 and day-16 MSCs, indicating the lack of surface marker selectivity for adipogenically differentiated cells (data not shown).

### Quantification of MSC adipogenic progression with the use of microplate assay with Nile red and DAPI

**Figure 4:**
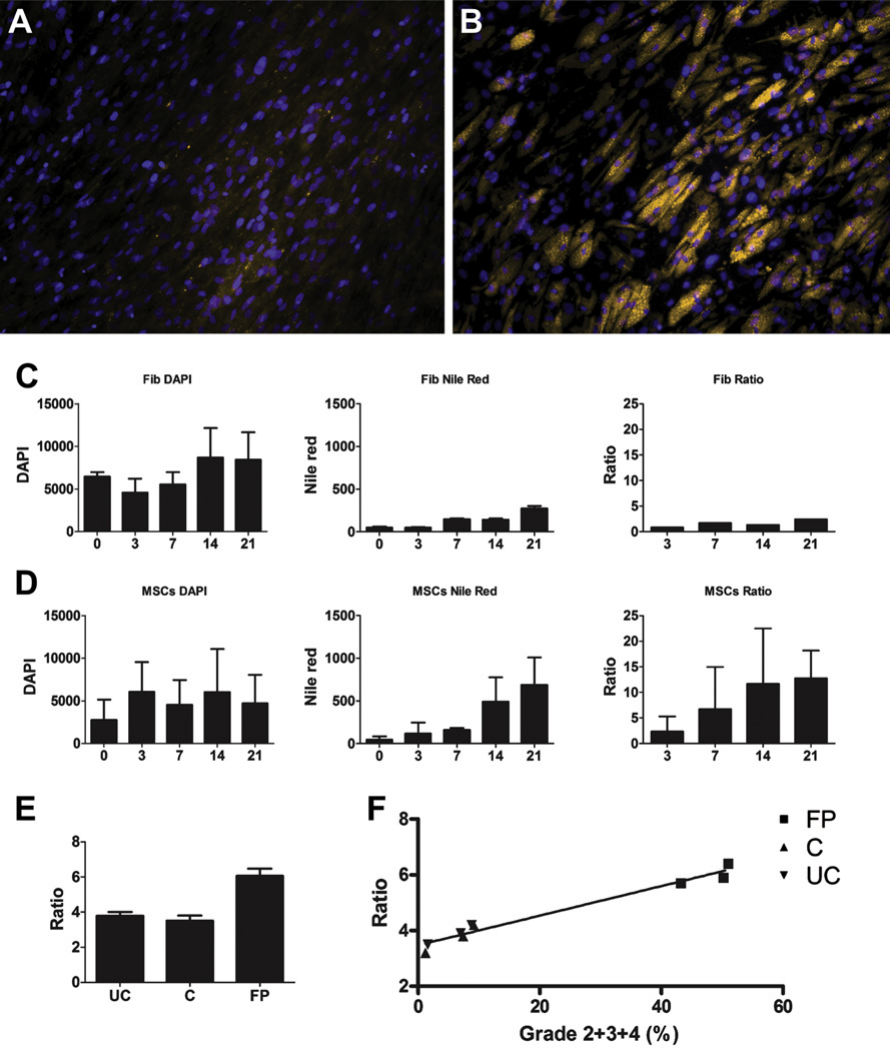
Nile red and DAPI staining of MSCs. MSCs and fibroblasts were grown for 0, 3, 7, 14 and 21 days in adipogenic medium and stained with Nile red dye and DAPI. Representative images of (A) fibroblasts and (B) MSCs on day 21 are shown. Intracellular lipid droplets in cells stain yellow/gold, and the nucleus is stained blue. Images shown are ×200 magnification. (C) Levels of fluorescence for DAPI and Nile red were measured for fibroblasts cultured in adipogenic medium. Error bars shown are ± standard deviation (deviation between replicate wells). (D) Levels of fluorescence for DAPI and Nile red were calculated for BM MSCs 1–4 cultured in adipogenic medium, and the mean values normalized to day 0 shown. Error bars shown are ± standard deviation (deviation between donors). (E) Adipogenesis of MSCs isolated from umbilical cord (UC), knee cartilage (C), knee fat pad (FP) and bone marrow (BM) on day 21 of culture. Error bars shown are ± standard deviation (n = 3 donors each). (E) Correlation between Nile red/DAPI ratios and grades 2+3+4 semiquantitative scoring. These methods exhibited significant levels of correlation (*r* = 0.983, *P* < 0.0001).

A Nile red–based fluorescence microplate assay was next evaluated for its suitability to measure adipogenesis, with DAPI staining for DNA used to account for cell proliferation/apoptosis (27,33). MSCs or fibroblasts were cultured in adipogenic medium in wells of a 48-well plate; DAPI and Nile red readings were taken on days 0, 3, 7, 14 and 21 after induction. Representative fluorescence microscopy images of fibroblasts and MSCs on day 21 are shown (Figure 4A and 4B, respectively).

To determine whether adipogenesis could be measured using Nile red only, levels of fluorescence for DAPI alone and Nile red alone were first measured. Initially, levels of DAPI staining decreased before a gradual increase in fibroblasts (suggesting initial apoptosis before recovery and proliferation) but fluctuated in MSCs after day 3 (Figure 4C,D). Nile red staining in fibroblasts remained low (Figure 4C) and, not surprisingly, showed a greater increase in MSCs (Figure 4D). The differences in Nile red accumulation in MSCs were on average 3-fold (day 7/day 0 ratio) and 4-fold (day 21/day 0 ratio).

Nile red fluorescence in MSCs was next normalized to DAPI staining to account for MSC proliferation/apoptosis, and a ratio of Nile red/DAPI was used to measure adipogenesis. Nile red to DAPI ratios demonstrated minimal adipogenesis in fibroblasts (Figure 4C) but an increase in adipogenesis in MSCs (Figure 4D). The differences in Nile red/DAPI ratios in MSCs were on average 7-fold (day 7/day 0 ratio) and 13-fold (day 21/day 0 ratio).

Both Nile red microplate assays and morphological scoring with oil red significantly correlated with each other for all donors and all time points tested (*r* = 0.931, *P* ≤ 0.0001). We finally evaluated congruency between these two assays through the use of MSCs derived from a highly adipogenic tissue [knee fat pad (25,40)] and poorly adipogenic tissues [knee cartilage (25) and umbilical cord (41–43)]. Consistent with observations obtained using oil red staining (25), the results for Nile red/DAPI ratios showed that knee fat pad MSCs had higher levels of adipogenesis compared with cartilage or umbilical cord–derived MSCs (Figure 4E). Regardless of the MSC tissue source used, the correlation between the oil red and Nile red assays was very good (*r* = 0.983, *P* < 0.0001) (Figure 4F).

## Discussion

There are currently more than 120 MSC-based clinical trials aimed to treat a number of human diseases including immune-related and auto-immune diseases as well as severe myocardial ischemia, bone defects, osteoarthritis or liver disease (clinicaltrials.gov). More recently, adipose tissue–derived MSC sources have become popular because of ease of access and harvesting of this tissue (more than 40 trials). Furthermore, the stromal vascular fraction (SVF) from lipoaspirates without the MSC isolation by culture amplification has attracted increased interest as a therapeutic product, presumably because of its low cost and ease of manufacture (44,45). However, concerns may exist relating to purity of these preparations and possible contamination with maturing fat lineage cells that can potentially be immunogenic (13,14,46). This necessitates the development of new tools to control the quality of manufactured MSC and SVF batches with respect to the presence of contaminating fat lineage cells at different stages of maturation and the assessment of their contribution to clinical outcomes after implantation. In addition to assaying for fat cell contamination, another viable application of adipogenic cell assays is the prediction of the development of undesirable cell phenotypes. With the increasing number of commercial products based on allogeneic MSCs, preliminary screening of MSC multipotentiality and adipogenic propensity in particular could be used to negatively select against the most adipogenic MSC donors.

With these considerations in mind, the aim of this study was to compare previously proposed independent methods for measuring MSC adipogenesis and to develop a reliable assay with well-defined *in vitro* conditions. In the present study, we report both advantages and disadvantages of the four methods tested (Table I) and particularly highlight (i) the utility of flow cytometry for dissecting cell-to-cell heterogeneity within the same differentiating culture, with a potential to sort less and more differentiated subpopulations and (ii) the advantage of a Nile red–based microplate assay to provide rapid measurements of both MSC adipogenesis and proliferation/apoptosis in differentiating adipogenic cultures.

Oil red dye is a member of the Sudan lysochrome family of dyes and has been used for more than 50 years as a stain for neutral triglycerides, phospholipids and cholesterol esters (47). Many studies used oil red to measure adipogenesis but made no attempt to quantify levels (3,48,49). Zimmerlin *et al.* (26) used a system that counted cells with at least five large oil red vesicles under low-power field microscopy (×10 objective and five to eight fields).

Other studies quantified adipogenesis by removing oil red from within the lipid vesicles with isopropanol and measuring the absorbance at 510 nm (50). The latter method, however, does not give any indication as to cell number or average levels of lipid per cell; in addition, the absorbance of samples is subject to variation caused by the degradation of isopropanol over time (isopropanol is a volatile organic solvent). Another method, the assessment of triglyceride content, can be performed after extraction of triglyceride from the cell by DMSO and then quantification through the use of enzyme-based kits and absorbance measurements (51). This method also has some of the same issues that extracting oil red from stained cells with isopropanol has in that it is difficult to determine cell number or average levels of lipid per cell.

In our experiments, we noted that not all cells within the same adipogenic culture differentiated at the same rate, which could be due to differing levels of pre-commitment between seeded MSCs in different donors and/or cell density, as suggested previously (22). Additionally, large donor-to-donor variability was observed with all assays used. Other studies have also commented on the donor-to-donor variability and heterogeneous nature of MSC populations derived from BM (52) or adipose tissue (53).

For q-PCR, two genes associated with adipogenesis were selected: PPAR-γ and the late marker of adipogenesis, FABP4. Both of these transcripts have been shown to increase in adipogenesis. PPAR-γ is a nuclear receptor, which, on stimulation with ligand, binds to retinoid-X receptor, and this complex can bind to DNA and initiate transcription (21). The exact factors involved in differentiation into mature adipocytes are not currently fully understood, but many different genes have been reported to be involved. These genes include CAAT/enhancer binding protein-α (C/EBPα), FABP4, insulin-like growth factor binding protein 2 (IGFBP2), resistin, adiponectin and lipoprotein lipase, which simulates fatty acid binding and sequestering in mature adipocytes (22,54,56). FABP4 was chosen as the most selected late marker of adipogenesis (22); it is also known as fatty acid binding protein (aP2) in other mammals and is involved in the metabolism and intracellular transport of fatty acids in adipose cells. It is able to bind the fatty acids with a high affinity and relocalize them to other parts of the cell (57) including to lipid droplets for storage, the endoplasmic reticulum for signaling, the mitochondria or peroxisome for oxidation and to the nucleus for the control of lipid-mediated transcriptional programs via nuclear hormone receptors, or other transcription factors that respond to lipids (58).

In contrast to PPAR-γ expression, which, under our experimental conditions, displayed only small changes, FABP4 expression increased by 2–3 orders of magnitude by the end of differentiation time course. Sole measurement of PPAR-γ expression by q-PCR was unlikely to be sufficient to monitor adipogenic progression in MSCs, whereas FABP4 expression increased most dramatically, consistent with previous findings (31), and correlated very well with microscopic fat accumulation in MSCs (*r* = 0.989, *P* < 0.0001). Our findings correlate well with the study of Boucher *et al.* (31). These investigators used a q-PCR assay to routinely characterize MSC differentiation into adipocytes, mainly for media potency evaluation, shortening the length of time required to 7 days. In their study, FABP4 upregulation was observed as early as day 3 after induction, with expression persisting throughout longer periods of differentiation, in agreement with our findings. Studies in cultures of adipose-derived MSCs have reported similar patterns of expression, in which PPAR-γ increased by a smaller order of magnitude than did FABP4 (59,60). This pattern of expression would correlate with FABP4 being transcriptionally activated by PPAR-γ itself (15). Our flow cytometry findings showed lower (∼6-fold) FABP4 upregulation (at the protein level) by day 21, demonstrating that mRNA levels do not directly translate into protein levels. The correct choice of reference gene(s) to be used when performing q-PCR is another issue that must be taken into account when designing protocols with the use of this technology (61).

In comparison to other methods, relatively few studies have used flow cytometry to track MSC adipogenesis (18,20,23,24,35–37). This is surprising because flow cytometry provides information on intensity of staining, size of cells, their granularity as well as cell number. We observed a gradual accumulation of more granular cells as the time course progressed, correlating with increasing intracellular FABP4 levels in these MSCs. Nile red dye has previously been used in flow cytometry and microscopy to stain adipocytes derived from murine (23,35,37,62,63) and human (18) MSCs. It is a selective fluorescent stain for intracellular lipid droplets emitting at 528–570 nm. When Nile red staining for lipids was assessed by flow cytometry, an increase in intracellular Nile red fluorescence, similar to FABP4 shift, was noted as the time course progressed, with more granular cells increasing in number and gaining higher amounts of Nile red fluorescence compared with less granular cells. This is in agreement with previous studies that demonstrated the appearance of a new population of cells with increased orthogonal light scattering (side scatter) over time (64,65). We noted, however, that low-SSC cells accumulated lipid at the same rate as high-SSC cells, suggesting that less granular cells were not totally “resistant” to adipogenesis. Notably, all MSC surface markers tested failed to distinguish between undifferentiated and adipogenically differentiated MSCs or between MSCs and fibroblasts, as reported previously (39).

Our findings are in agreement with earlier studies (64,66) and more recent studies that have used flow cytometry to sort adipocytes from mixtures of ES-derived mesenchymal lineage cells (67). Altogether, these data show that flow cytometry is a useful tool for tracking adipogenesis, additionally allowing the isolation of less and more differentiated cells from the same differentiating culture by fluorescent activated cell sorting (FACS). Because flow cytometry is a powerful multiparameter technique, gating strategies using FABP4 or Nile red positivity can be further used to discover novel surface markers of adipogenically committed cells. However, we found a loss of the most mature, high-SSC between day 14 and day 21. This may have been due to the increased buoyancy of the intracellular lipid droplets in the most mature adipocytes, causing their loss during the wash steps. The high lipid content of these adipocytes could also alter the strength of the cell membrane, resulting in cells being more fragile and prone to bursting. Therefore, a considerable disadvantage of flow cytometry is an underestimation of the proportion of the most mature adipocytes (Table I).

Visual analysis using oil red staining showed heterogeneity within the same adipogenesis-driven MSC cultures, with a subset of the cells failing to differentiate into fat cells. This is in contrast to what was observed with the use of flow cytometry, in which gradual fat accumulation by all cells was detected. This could be a result of the subjective nature of the oil red grading system, which relies on visual recognition and grading by the researcher.

The quantitative assay with the use of intracellular Nile red fluorescence for lipid accumulation and DAPI staining of cell nuclei for normalization was first described by Jaiswal *et al.* (33). In our experiments, MSCs stained with DAPI have varied fluorescence over the time course, demonstrating that some cells continued to proliferate even when others were differentiating at various levels. We confirmed that adipogenesis can be assessed using a single dye (18,23,50,62,63), but, to account for proliferation of cells, a ratio with the use of Nile red and DAPI together was preferable. The use of a sole Nile red dye to measure lipid content may underestimate or overestimate adipogenesis because there is no compensation for proliferation or apoptosis. To its other advantage, the fluorescent microplate assay can also provide visual verification of adipocyte content per cell. In all assays evaluated, fibroblasts exhibited very low levels of adipogenesis. The same was evident for cartilage-derived cells, as previously reported (25), and the opposite was true for fat pad–derived cells, demonstrating that the Nile red assay was robust regardless of the MSC tissue source used.

In summary, this study explored a combination of methods to study MSC differentiation toward adipogenesis. As a result, flow cytometry for FABP4 or Nile red staining is proposed to be useful to study *in vitro* differentiation of MSC cultures at the single-cell level and potentially for the assessment of mature adipose-lineage cell contamination in SVF preparations intended for therapy. It can also be applied to the study of MSC maturation toward the fat tissue in *in vivo* investigations, particularly in relation to immunogenic effects associated with fat tissue formation from implanted MSCs. The precedent for this exists for bone neotissue, whereby its origin and maturation status *in vivo* could be established after tissue excision and enzymatic digestion followed by flow cytometry (68). In relation to a microplate assay measuring Nile red/DAPI ratios, we propose its use for defining the cutoff point below which putative MSC cultures are considered to lack adipogenic potential, an important measure of MSC multipotentiality. At present, the International Society for Cellular Therapy position statement defining MSCs advises qualitative assessment only of their adipogenesis (69), resulting in confusion as to whether skin fibroblasts or cultured chondrocytes (and other poorly adipogenic adherent cells) can be defined as MSCs (39,70–72). A joint effort from the broader scientific community will be required to validate the robustness of such an assay across different research centers.

## Figures and Tables

**Table I:** Advantages and disadvantages of different assays for the evaluation of MSC adipogenesis.

Method	Assay time	Number of cells needed	Subjectivity	Added value	Overview including disadvantages
Semiquantitative scoring with oil red	Short (one-half day): staining 45 min, scoring depends on the number of samples	500 cells/coverslip in triplicate	Semiquantitative Highly subjective	Information on cell-to-cell heterogeneity in fat content per cell within the same culture	Subjective but very simple and inexpensive
q-PCR for FABP4	Long (1 day): RNA extraction: 30 min cDNA synthesis:2–3 h PCR: ∼2 h	10^4^ cells per RNA extraction	Semiquantitative [relative quantification requires normalization to an appropriate reference gene (62)] Less subjective	Other early and late adipogenesis-related transcripts (22,55–57) can be added at minimal cost	Fairly quantitative at the transcript level but time-consuming and requires validation at the protein level. No information on cell-to-cell heterogeneity. Optimization time (could be reduced by use of a Taqman assay).
Flow cytometry with Nile red	Short (one-half day) Harvesting cells: 30 min to 1 h Staining: 30 min to 1 h Flow cytometry: 1 h plus (amount of time required depends on number of samples being run and number of cells/sample)	10^5^ cells per test	Highly quantitative Less subjective	Permits identification and purification of more and less-differentiated subsets by FACS and its multiparameter nature can lead to discovery of novel surface markers associated with adipogenesis	Highly quantitative, rapid and inexpensive. Potential for underestimation of the most differentiated (hence, buoyant) fat cells as the result of their loss through trypsinization/washing steps. Lack of gaussian fluorescence profiles. Possibility lipid vacuoles from broken cells could be counted as cells; accurate gating strategy needed.
Fluorescent microplate assay with Nile red/DAPI	Short (less than one-half day) Staining: 1 h Reading: 30 min	4 × 10^4^ cells in triplicate	Highly quantitative Least subjective	High throughput Extra information on proliferation/apoptosis (DAPI only)	Highly quantitative, rapid and inexpensive. Can be combined with semiquantitative scoring with the use of fluorescent microscope.
